# Toxicity of Methylated Bismuth Compounds Produced by Intestinal Microorganisms to *Bacteroides thetaiotaomicron*, a Member of the Physiological Intestinal Microbiota

**DOI:** 10.1155/2011/608349

**Published:** 2011-09-28

**Authors:** Beatrix Bialek, Roland A. Diaz-Bone, Dominik Pieper, Markus Hollmann, Reinhard Hensel

**Affiliations:** ^1^Department of Microbiology I, University of Duisburg-Essen, 45141 Essen, Germany; ^2^Department of Instrumental Analytical Chemistry, University of Duisburg-Essen, 45141 Essen, Germany; ^3^Department of Environmental Analytical Chemistry, University of Duisburg-Essen, 45141 Essen, Germany

## Abstract

Methanoarchaea have an outstanding capability to methylate numerous metal(loid)s therefore producing toxic and highly mobile derivatives. Here, we report that the production of methylated bismuth species by the methanoarchaeum *Methanobrevibacter smithii*, a common member of the human intestine, impairs the growth of members of the beneficial intestinal microbiota at low concentrations. The bacterium *Bacteroides thetaiotaomicron*, which is of great importance for the welfare of the host due to its versatile digestive abilities and its protective function for the intestine, is highly sensitive against methylated, but not against inorganic, bismuth species. The level of methylated bismuth species produced by the methanoarchaeum *M. smithii* in a coculture experiment causes a reduction of the maximum cell density of *B. thetaiotaomicron*. This observation suggests that the production of methylated organometal(loid) species in the human intestine, caused by the activity of methanoarchaea, may affect the health of the host. The impact of the species to reduce the number of the physiological intestinal microbiota brings an additional focus on the potentially harmful role of methanoarchaea in the intestine of a higher organism.

## 1. Introduction

Biomethylation of metals and metal(loid)s is an important process which increases the mobility, bioavailability, and toxicity of these elements. Anaerobic microorganisms, in particular methanoarchaea, show the greatest versatility regarding the spectrum of elements that they methylate [1]. Considering human health, the biotransformation of harmless metals, such as bismuth, by the human intestinal microbiota is a highly relevant process. Due to the low toxicity of metallic bismuth and its inorganic salts, bismuth has been classified as a “green element” [2]. Bismuth is therefore widely used in a variety of applications such as cosmetics, catalysts, industrial pigments, and ceramic additives [3]. Bismuth is, however, associated with several adverse reactions such as encephalopathy, renal failure, and even cases of death in the 70s and 80s [4, 5]. It has been suggested that derivatives of this metal may be responsible for these damages. 

Our recent studies have shown that, after ingestion of inorganic bismuth, the intestinal microbiota, in particular methanoarchaea, are capable of methylating inorganic bismuth to soluble paritally methylated compounds like monomethyl- (MMBi-) and dimethylbismuth (DMBi) as well as volatile trimethylbismuth (TMBi) [6–10]. TMBi is characterized by a higher volatility and hydrophobicity in comparison to inorganic bismuth and can therefore be easily distributed inside the human body and is able to pass the blood-brain barrier [11]. In mice fed with chow containing colloidal bismuth subcitrate (CBS), elevated concentration of TMBi in their blood and enrichment of Bi in several organs were detected [12, 13]. While the direct impact of the toxic methylated compounds on the physiology of the human body has already been addressed [14], the indirect negative effect of these compounds on the human health by inhibition of the growth of members of the beneficial intestinal microbiota has been little investigated so far. 

To overcome this neglect, the influences of methylated bismuth species are investigated for a prominent member of this microbiota, notably for *Bacteroides thetaiotaomicron *[15]. This gram-negative anaerobic bacterium has some advantages for the host. For example, it has some versatile digestive abilities which benefit higher organisms [16]. Additionally, this microbe is important for the defense against the adverse activities of pathogenic microorganisms and guarantees the integrity of the intestine epithelium, thus ranking among the so-called physiological intestinal microbiota [17, 18]. In a previous study, this organism is found to be sensitive to permethylated bismuth, (CH_3_)_3_Bi [1]. In this study, the toxic effects of partially and fully methylated bismuth derivatives on *B. thetaiotaomicron* were studied in more detail using an *in vivo*-like coculture system with the methanoarchaeum *M. smithii* as the producer of TMBi.

## 2. Results and Discussion

### 2.1. Influence of Colloidal Bi Subcitrate (CBS) on Growth of Cultures of *B. thetaiotaomicron*

**Figure 1:**
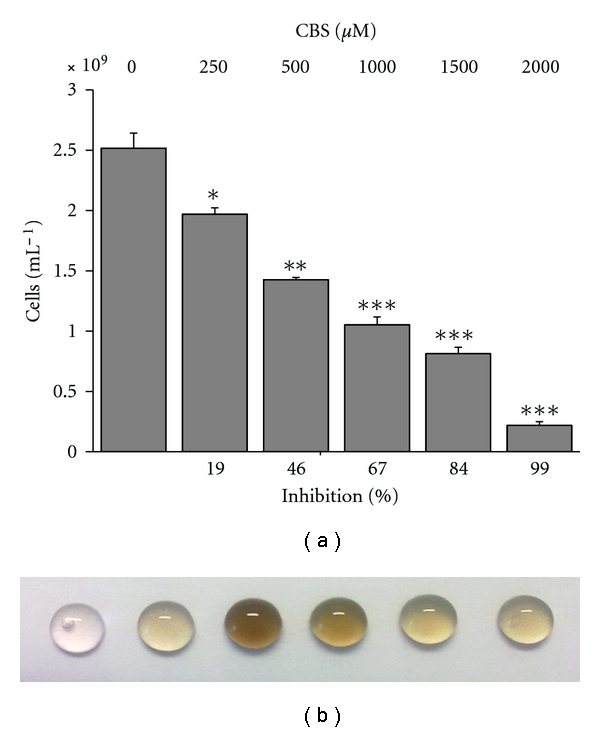
Growth inhibition of *B. thetaiotaomicron* 48 h cultivation after addition of CBS (*n* = 3). The addition of CBS to the growing *B. thetaiotaomicron* cultures (a) resulted in an inhibition of the maximal cell density in the stationary phase at 48 h after the addition of CBS in a concentration-dependent manner.  Figure 1(b) shows a black precipitation in 20 *μ*L droplets from the cultures.

In all experiments concerning the toxic effects of bismuth species, we evaluated the reduction of the maximal cell density at the stationary phase upon the application of the various drugs in the late exponential phase. This was taken as a measure for the antimicrobial activity of the compounds. As shown in Figure 1(a), the addition of 250 *μ*M up to 2000 *μ*M CBS to growing cultures of *B. thetaiotaomicron* resulted in a reduction of maximal cell density in the stationary phase. With increasing concentration, stronger growth inhibition is observed with a minimal inhibitory concentration (MIC_50_) of about 500 *μ*M CBS.

The addition of CBS resulted in a black precipitation of bismuth sulfide (Figure 1 (b)). This is due to the reaction with hydrogen sulfide produced from the anaerobic metabolism of *B. thetaiotaomicron*. The most intensive precipitation was observed at 500 *μ*M similar to the MIC_50_. Furthermore, the increasing inhibition of CBS on the growth of *B*. *thetaiotaomicron *resulted in a reduction of the sulfide precipitate.

### 2.2. Influence of the Methylated Derivatives of Bismuth

**Figure 2:**
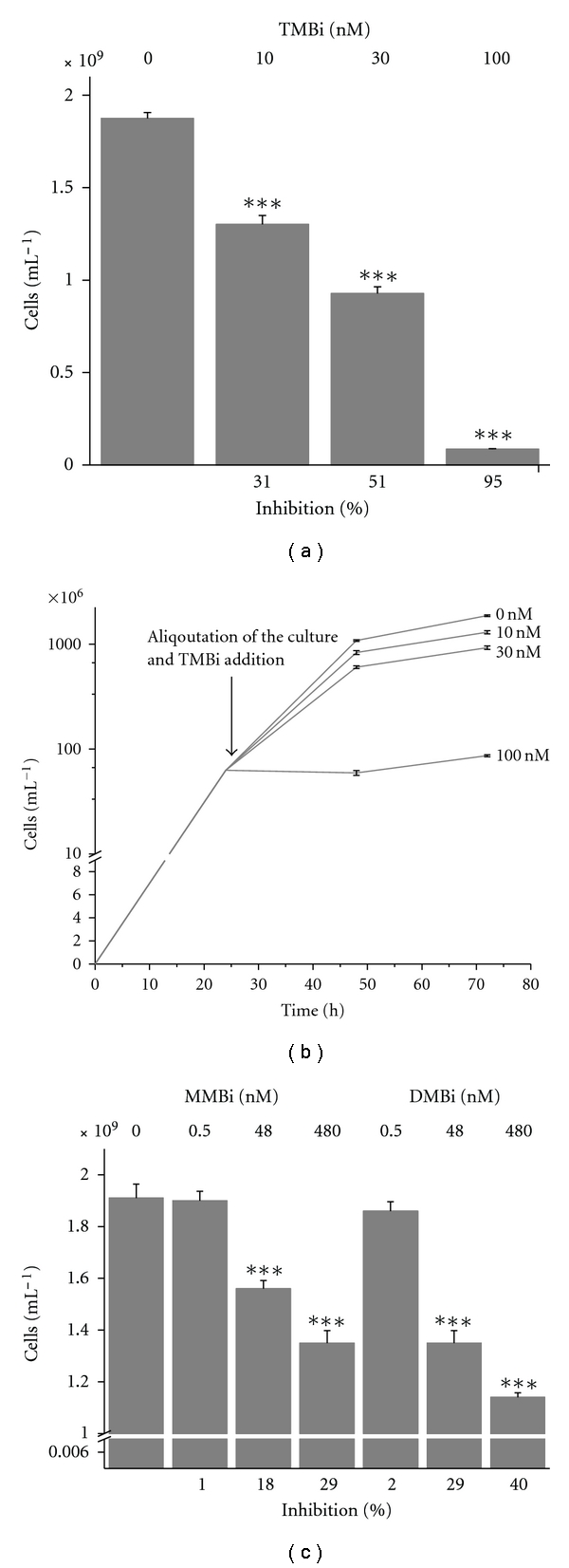
Growth inhibition of *B. thetaiotaomicron* 48 h after exposure of methylated bismuth species (*n* = 3). The addition of TMBi (a) to the headspace or of nonvolatile methylated bismuth species MMBi and DMBi (c) to the liquid phase of growing *B. thetaiotaomicron* cultures resulted in a growth reduction in a concentration-dependent manner.  Figure 2(b) shows the growth curve in the presence of TMBi. The culture was separated in aliquots in the late exponential phase and was exposed to different TMBi concentrations. The cell counts were determined after 48 h at the stationary phase.

Similar growth inhibition effects were observed upon the addition of the methylated bismuth compounds monomethylbismuth, dimethylbismuth, and trimethylbismuth, however, at significantly lower concentrations (Figures 2(a) and 2(b)). The concentrations for the observed growth inhibition were within the nM range (MIC_50_, 30 nM TMBi) and therefore four orders of magnitude lower compared to bismuth subcitrate (500 *μ*M). In case of TMBi, an addition of up to 30 nM to the headspace resulted in an inhibition of about 50% and therefore at a concentration which is slightly higher than that in a previous study with *B. thetaiotaomicron *(MIC_50_, 17 nM TMBi) [1]. This is presumably due to the addition of TMBi together with the inoculum. Interestingly, the GC-ICP-MS measurement of the headspace of *B. thetaiotaomicron* after the exposure time indicated that TMBi rapidly degrades in the presence of *B. thetaiotaomicron* cultures. Thus, the question arose whether the toxicity of TMBi is attributed to TMBi itself or to its partly methylated degradation products. Therefore, the toxicity of partially methylated Bi-derivatives on *B. thetaiotaomicron *was assessed.

### 2.3. Differentiation between the Inhibiting Effects of the Partially Methylated Bi-Derivatives

In experiments with partially methylated, soluble bismuth derivatives monomethylbismuth (MMBi) and dimethylbismuth (DMBi), inhibiting effects on the growth of *B. thetaiotaomicron* were observed at similar levels as those for TMBi (Figure 2(c)). After the addition of these derivatives within the exponential phase, the cell growth was reduced and did not reach the maximum cell density at the stationary phase compared to untreated control cultures. At a concentration of 48 nM, a significant growth inhibition is observed for both MMBi (18% reduction) and DMBi (29% reduction). While the toxicity of partially methylated Bi-derivatives is greater than that of inorganic bismuth, it is in a similar range, but in a lower range than that of TMBi.

### 2.4. Coculture of *B. thetaiotaomicron* with *M. smithii*

**Figure 3:**
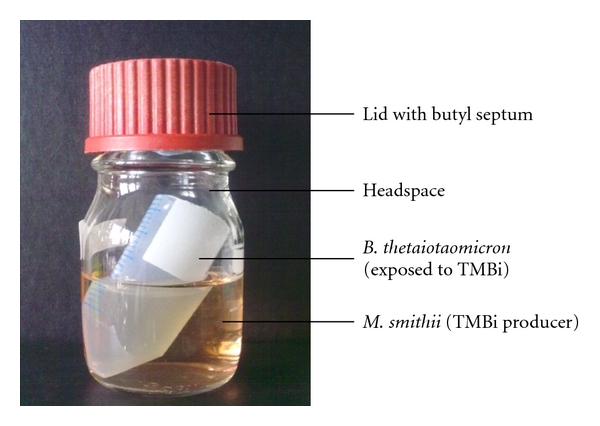
Design of the coculture system. The represented coculture system exhibited two separate liquid cultures under a common headspace. This design allowed the transfer of produced volatile TMBi from the culture of *M. smithii* to the culture of *B. thetaiotaomicron* over the common gas phase.

**Figure 4:**
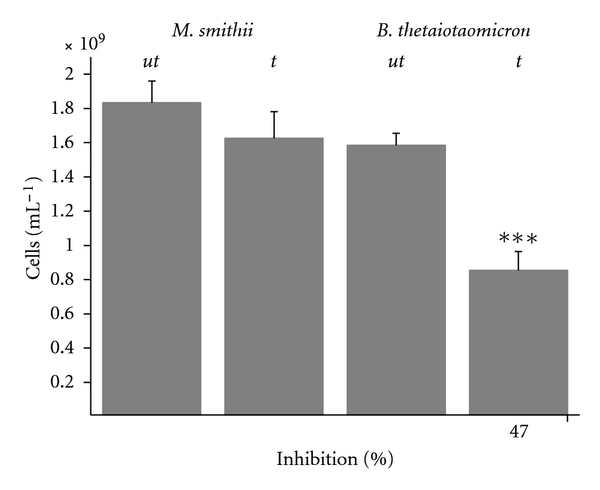
Reduction of the cell counts of *B. thetaiotaomicron* due to the production of volatile TMBi produced by *M. smithii* (*n* = 3).* M. smithii* and *B. thetaiotaomicron* were grown in the coculture system. CBS was applied at a concentration of 80 *μ*M to the culture of *M. smithii* in its late exponential phase. The TMBi production of *M. smithii*, during its 48 h incubation in the presence of CBS, results in a significant cell count reduction of *B. thetaiotaomicron* cultures (*t*) compared to untreated control (*ut*).

To confirm the relevance of the *in vitro* results for the situation *in vivo*, an *in vivo-*like coculture system was constructed (Figure 3). The design presents two separate fluid cultures under a common headspace in order to study the toxic effects of TMBi produced by *M. smithii* on *B. thetaiotaomicron*. CBS was applied to the culture of* M. smithii* in the late exponential phase (80 *μ*M), and the growth behavior of *B. thetaiotaomicron *was followed over 48 h. As shown in Figure 4, the growth of* B. thetaiotaomicron* was reduced to approximately half of the density of the untreated control.

In order to verify that the production of TMBi is the reason for the growth reduction, we measured the concentration of the evolved Bi-derivative TMBi in the headspace over 48 h. While in the headspace of isolated *M. smithii* cultures TMBi is found [1], no volatile TMBi was detected in the coculture system due to the rapid degradation of TMBi over the *B. thetaiotaomicron* culture medium as described above. Thus, an alternative approach was developed based on passive TMBi chemotrapping using silver nitrate-coated silica beads analogous to a method recently introduced for the sampling of volatile arsenic and selenium species by Uroic et al. [19]. The *B. thetaiotaomicron* culture located in the inner tube was replaced by the chemotrap and the bismuth content of the chemotrap after 48 h of incubation was analysed by ICP-MS; 1.64 ± 0.04 nmol Bi were trapped by AgNO_3_-coated silica beads corresponding to 33 nM TMBi in the gas phase, which is quite similar to the MIC_50_ of TMBi.

## 3. Conclusion

The present work confirms that the methanogens represent members of the intestinal microbiota with the hazardous capability to transform metal(loid)s into toxic methylated derivatives. The volatile organometal(loid) species formed do not only interact directly with the host's organ tissues (e.g., by increasing bioabsorption of bismuth [12] or by intoxification of mammalian cells [20]) but also indirectly. The indirect interaction inhibits the beneficial microbiota in its capacity to help with optimal digestion of complex food and protect the intestinal epithelium. We showed in our research that both volatile TMBi as well as nonvolatile partially methylated derivatives interact with members of the physiological intestinal microbiota. The measurements of the concentration of TMBi in the intestines of mice fed with CBS-containing chow (approximately 8–10 nM, unpublished data) suggest that the produced amount of TMBi is sufficient for this negative interaction, which ends up in a strong inhibition of this microbiota.

Unfortunately, nothing is known about the molecular mechanisms of these phenomena. In addition, the higher hydrophobicity of methylated species will increase the mobility of these compounds and allow them to interact with the cell membrane, enter, and damage the cells. An interaction with inner components of the cell cannot be excluded. Bi^3+^ ions may interact with essential proteins of *B. thetaiotaomicron *and methylated bismuth derivatives could cause methylation reactions with macromolecules, as shown for arsenic [14]. Taking into consideration that methylated bismuth species are by orders of magnitude more toxic than inorganic bismuth and that toxicity decreases with a decreasing degree of methylation together with the instability of TMBi, it seems plausible to assume that the toxicity of TMBi as well as that of the partly methylated species is due to a methyl transfer during degradation of these highly unstable species. 

Considering the high potential for negative effects of methanogens on higher organisms, their harmful nature seems to be beyond question. But are the methanogens really examples for true pathogenic archaea? “Pathogenic” means that the organism in question deliberately damages the host for the advantage to reproduce and spread. But does this apply to the methanogens? In a recent study, we have proven that the methylation of metal(loid)s by *Methanosarcina mazei* is not a deliberate detoxification mechanism in analogy to the ArsM system for arsenic. Instead it is a result of the side reaction of methanogenic cofactors (Thomas et al., submitted [21]). Methanogens therefore affect the host merely because of a coincidence of the methanogenesis intermediates and the formation of metal(loid) derivatives. Thus, a special advantage from the production of these harmful metal(loid) derivatives does not seem obvious.

## 4. Material and Methods

### 4.1. Standard Cultivation

Growth experiments with *B. thetaiotaomicron* (DSMZ No. 2079) and *M. smithii* (DSMZ No. 861) were performed under strict anaerobic conditions (gas phase: H_2_/CO_2_ (80 : 20)) at 37°C in serum bottles with a total volume of 120 mL (50 mL fluid, 70 mL headspace). The following complex growth medium was used for both *B. thetaiotaomicron* and *M. smithii*: 0.5 g KH_2_PO_4_, 0.4 g MgSO_4_× 7 H_2_O, 0.4 g NaCl, 0.4 g NH_4_Cl, 0.05 g CaCl_2_× 2 H_2_O, 1 g C_2_H_3_KO_2_, 8 g NaCOOH, 4 g NaHCO_3_, 6 g bacto brain heart infusion, 2 g yeast extract, 6 g peptone, and 10 mL SL-10 (DSMZ) per liter.

### 4.2. Coculture System

A coculture system with two separate liquid and one headspace phase is constructed by using a 100 mL glass bottle with thick butyl septum (Schott AG, Mainz, Germany) and 30 mL greiner tubes (40 + 10 mL fluid, 50 mL headspace).

### 4.3. Quantification of Cells

Cultivated cells are counted with a Thomson counting chamber in moderate dilution.

### 4.4. Addition of Bismuth

Cultures of *B. thetaiotaomicron* are aliquoted and different bismuth species were added within the late exponential growing phase (1 × 10^8^ cells·mL^−1^). Inorganic and soluble bismuth species were added to the liquid phase of *B. thetaiotaomicron*. Volatile TMBi was applied to the headspace.

### 4.5. Analysis of Volatile Metal(loid) Derivatives

Volatile TMBi was analyzed by a modified purge and trap gas chromatograph system [22] coupled to an ELAN 6000 (PerkinElmer) inductively coupled plasma mass spectrometer (P&T-GC-ICP-MS) as described elsewhere. The identification of volatile metal(loid) compounds based on GC-ICP-MS boiling-point retention correlation was verified by using parallel molecular and elemental mass spectrometry (GC-EI-MS/ICP-MS) as described previously [23].

### 4.6. Chemotrapping of Volatile TMBi

Volatile TMBi was trapped by using silica beads coated with silver nitrate modified from Uroic et al. [19]. These beads were placed over night in a 1% silver nitrate solution and then dried at 40°C. 1 g of these coated silica-beads was placed in the coculture system the inner tube instead of *B. thetaiotaomicron*. After 48 h of incubation, the production of TMBi by *M. smithii* was determined by extraction of the beads with 5% HNO_3_, and analysis by ICP-MS after sterile filtration.

### 4.7. Synthesis of Methylated Bismuth Compounds

The methylated bismuth compounds were synthesized in a two-step process. In a first step, trimethylbismuth was prepared in a method adapted from Marquardt [24]. In brief, a Grignard solution was prepared by adding methyl iodine to magnesium chippings in diethyl ether. Trimethylbismuth was isolated by fractionated distillation. In a second step, trimethylbismuth was solved in diethyl ether and bismuth bromide was added in a molar ratio of 2 : 1 of BiBr_3_ and (CH_3_)_3_Bi to yield (CH_3_)BiBr_2_ or in a 1 : 2 ratio to yield (CH_3_)_2_BiBr, respectively. Bismuth cysteine and methyl bismuth cysteine were prepared by adding bismuth(III) bromide (BiBr_3_) or methylated bismuth bromide ((CH_3_)BiBr_2_), respectively, to a saturated solution of L-cysteine in ultrapure laboratory water. The molar bismuth ratio of the bismuth compound to L-cysteine was 1 : 2. The mixture was stirred at room temperature (20°C) in an inert argon atmosphere. For isolation of these compounds we added small amounts of methanol until precipitation of a yellow solid. The crystalline product was subsequently filtered through a fiberglass filter and dried in vacuum desiccators. Verification of the standards was performed by derivatization by ethylation and analysis by GC-MS as reported previously [25].

### 4.8. Statistical Evaluation

The significance of the cell numbers were analyzed using a pair-sample *t*-test. At the 0.05 level, the difference of the population means was significantly different with the test difference.
